# Using an Uncertainty-Coding Matrix in Bayesian Regression Models for Haplotype-Specific Risk Detection in Family Association Studies

**DOI:** 10.1371/journal.pone.0021890

**Published:** 2011-07-15

**Authors:** Yung-Hsiang Huang, Mei-Hsien Lee, Wei J. Chen, Chuhsing Kate Hsiao

**Affiliations:** 1 Institute of Epidemiology and Preventive Medicine, National Taiwan University, Taipei, Taiwan; 2 Department of Mathematics and Computer Science Education, Taipei Municipal University of Education, Taipei, Taiwan; 3 Department of Public Health, College of Public Health, National Taiwan University, Taipei, Taiwan; 4 Research Center for Genes, Environment, and Human Health, College of Public Health, National Taiwan University, Taipei, Taiwan; 5 Bioinformatics and Biostatistics Core, NTU Center for Genomic Medicine, National Taiwan University, Taipei, Taiwan; Aarhus University, Denmark

## Abstract

Haplotype association studies based on family genotype data can provide more biological information than single marker association studies. Difficulties arise, however, in the inference of haplotype phase determination and in haplotype transmission/non-transmission status. Incorporation of the uncertainty associated with haplotype inference into regression models requires special care. This task can get even more complicated when the genetic region contains a large number of haplotypes. To avoid the curse of dimensionality, we employ a clustering algorithm based on the evolutionary relationship among haplotypes and retain for regression analysis only the ancestral core haplotypes identified by it. To integrate the three sources of variation, phase ambiguity, transmission status and ancestral uncertainty, we propose an uncertainty-coding matrix which combines these three types of variability simultaneously. Next we evaluate haplotype risk with the use of such a matrix in a Bayesian conditional logistic regression model. Simulation studies and one application, a schizophrenia multiplex family study, are presented and the results are compared with those from other family based analysis tools such as FBAT. Our proposed method (Bayesian regression using uncertainty-coding matrix, BRUCM) is shown to perform better and the implementation in R is freely available.

## Introduction

Many genetic studies of complex diseases are interested in detecting associations between genetic markers and disease status. To evaluate the strength of such association, a regression approach may be adopted and applied to family haplotype data. Advantages of this regression framework include the ability to estimate and test the association, and its flexibility in accommodating not only individual information, but also gene-gene and gene-environment interactions. In addition, as compared with single-point SNP analysis, consideration of haplotypes as markers may provide better biological interpretation, and the selection of a family study design may lead to identification of susceptibility alleles inherited among family members.

Difficulties arise, however, with family haplotype data in regression models. One difficulty concerns the determination of haplotype phase, which involves uncertainty in inferring haplotypes from genotype data, and in differentiating between transmitted and non-transmitted haplotypes inherited from parents. Two groups of remedies have been suggested in previous research. The first, originally used in case-control studies [1]–[3], replaced the unknown phase with a maximum likelihood estimate or an expectation from an EM algorithm. For family data, Horvath and colleagues [4] considered weighted genotype scoring in tests with FBAT, and Purcell et al. [5] used the EM estimate in the free software WHAP. The second group of remedies, in contrast, included the set of all possible haplotype configurations compatible with the observed genotype, constructed the corresponding likelihood for each haplotype explanation, and then put weights on these likelihoods or log-likelihoods to establish a full likelihood function for case-control studies [6], [7]. Cordell et al. [8] gave a detailed comparison and review of these methods in two-stage analysis, under the assumption of a multiplicative model for case-control studies. For the family data here, we preserve the uncertainty in haplotype configurations with a rationale similar to that of the second group of remedies.

The second complexity encountered in association analysis is the large number of haplotypes available in the candidate region. This can result in a large number of degrees of freedom in statistical analysis and a phenomenon of sparsity in haplotype distribution. Many statistical methods have been proposed for dimension reduction, including dropping/grouping rare haplotypes, and clustering haplotypes based on their spatial relation or similarity in terms of an evolutionary relationship or length measure. Igo et al. [9] have provided an excellent review with many more references.

Because the analysis considered in this article is for family data, a preferred clustering algorithm should be able to track and manage the unknown haplotype phase, frequency, and transmission status simultaneously. Tzeng's [10] procedure accounted for the first two types of uncertainty. It defined the “age” of haplotype in terms of frequency, categorized the “generation” with the number of different components between two haplotypes, and weighted the clustering probability based on haplotype frequencies. Lee et al. [11] extended this procedure to family data by incorporating the transmission uncertainty in core haplotype assignment, and then combined it with a likelihood ratio test. We adopt this evolutionary-guided clustering idea and utilize a matrix containing all three types of uncertainty, in terms of probability, for haplotype compositions for each individual.

Another issue regarding the use of regression models for haplotype data is the specification of the design matrix when haplotype composition is considered as the covariate. Because each individual has two haplotypes, the sum of possibilities in haplotype assignment is a fixed constant, say 2. In other words, there exists collinearity among columns of the regression design matrix. Several researchers have suggested taking the most common haplotype as the reference to combat collinearity, and then focusing the inference on relative risks. Lin et al. [12] described a flexible coding when there exists a target haplotype for investigation, and demonstrated identifiability for regression parameters. In Bayesian analysis, prior specification on correlated covariates has attracted considerable attention, especially in the setting of Bayesian variable selection. Moreover, Soofi [13] showed that, when the prior variance is small relative to the variability in response, the difference in information for posterior inference is slight. Therefore, we employ only independent priors in the analysis. Alternatively, one could use the powered correlation prior or Zellner's *g*-prior to handle problematic collinearity [14].

In this study, under the regression framework with family data, we first match the affected child carrying the transmitted haplotypes to a pseudo-control child carrying the non-transmitted haplotypes. Next we formulate a regression setting under a Bayesian conditional logistic regression model with dichotomous disease status as the response variable. We propose in this model a design matrix whose entries represent the uncertainty in haplotype phase configuration, transmission, and clustering. Based on this Bayesian model, the haplotype specific risk can be evaluated as a posterior probability which takes haplotype uncertainty into account when only family genotype data are available.

## Methods

### Haplotype Coding with no Uncertainty

Consider 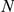 families, each with 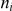 (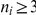, 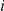 = 1, 2,…, 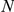) members, including an affected offspring, his/her parents, and any siblings. All of these participants are genotyped in the region of interest, where the number of available compositions of haplotypes 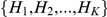 in this region is 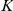. Among the four haplotypes from parents, two haplotypes are transmitted to the affected child and the remaining two non-transmitted haplotypes are included via a matched pseudo-control child. Let 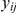 represent the dichotomous disease status, 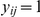 for case and 0 for normal. In such a matched case-control study, we consider for convenience the index 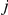 = 1 for the affected child, and 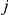 = 0 for the corresponding pseudo-control. In addition, let 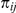 be the conditional probability that 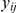 equals 1, 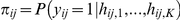, where 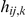 is the number of 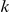-th haplotypes 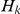 the child inherited from his or her parents. For instance, 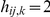 if this child inherited 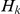 from both parents, 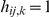 if 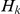 was inherited from either the paternal or maternal side, and 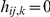 if the 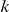-th haplotype does not provide any information regarding the transmission route; thus, 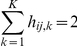. For this matched case and pseudo-control design, a conditional logistic regression model can be considered, and 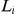, the likelihood for the 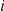-th family, can be directly written as
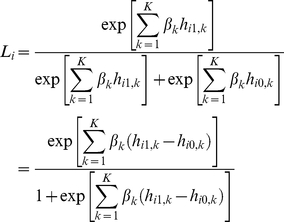
(1)where 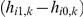 is, for the 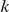-th haplotype, the difference in haplotype number between the affected child and the corresponding pseudo-control.

When there is no haplotype ambiguity, these 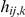 can be placed directly in a design matrix 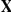, and then the inference of the corresponding coefficients 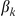 can be used to evaluate the strength of association, in terms of the logarithm of the odds ratio. To assess haplotype-specific risk when only genotype data are available, we propose another design matrix with coding for phase, transmission, and ancestry uncertainty.

### Haplotype Coding with Haplotype Phase Uncertainty

#### Uncertainty in Haplotype Explanation

When haplotype phase cannot be uniquely determined based on genotypes, particularly when parents' genotypes are missing, all possible configurations compatible with genotypes of parents and siblings can be inferred. In that case, 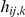 indicates the haplotype likelihood and can take any value between 0 and 2 with the same constraint that the summation of 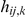 over 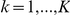 is 2.

Based on the observed genotypes of family members, a set 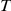 containing all possible combinations of transmitted and non-transmitted haplotypes can be derived. For instance, the set for the 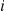-th family, consisting of three members in this example, is
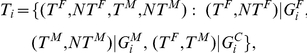
where 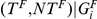 indicates the set of paternal haplotypes compatible with the observed paternal genotype 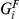, and 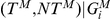 and 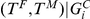 indicate the analogous explanations for the mother and the affected child, respectively; 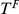 and 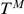 are the haplotypes transmitted from the father and mother, respectively; and 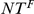 and 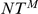 are the non-transmitted ones. When there are 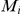 possible explanations for the 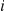-th family, the 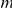-th (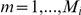) explanation component in 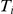 is denoted as a quadruple unit 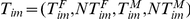. Its corresponding likelihood 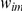 is proportional to the product of frequencies 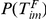, 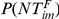, 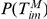 and 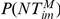, under the constraint that all likelihoods in 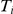 sum to 1. Therefore, if there are 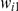, 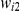, …, and 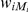 such likelihoods in 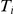, then for 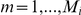,
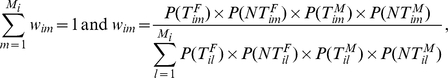
assuming independent sampling of haplotypes from the population.

For example, if the genotypes on two given loci are (1/2, 1/2) for the father, (−/−, 1/2) for the mother with the first genotype missing, and (1/1, 1/1) for the affected child, then the transmitted haplotypes from the father and mother along with the non-transmitted haplotypes (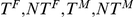) can be either (11, 22, 11, 12) or (11, 22, 11, 22). The uncertainty comes from the missing maternal genotype (−/−) of the first locus whose genotype can be either 1/1 or 1/2. Therefore, the haplotype phase of the pseudo-control can be either (22, 12) or (22, 22). Let *p*_1_ be the haplotype frequency for (12), and *p*_2_ for (22), then the conditional probability for phase (22, 12) is 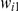 (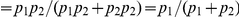) and 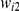 (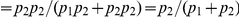) for (22, 22).

#### Uncertainty in Haplotype Transmission

Once the haplotype explanation set is defined and the uncertainty associated with each explanation is established, the next step is to determine the uncertainty regarding each transmitted haplotype. Under the assumption of additive haplotype effects, we construct for the case individual (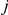 = 1) the haplotype weight 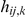 associated with 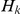. This weight includes both haplotype explanation uncertainty and haplotype transmission uncertainty:
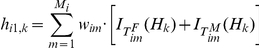
for 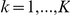. The above 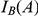 is an indicator function taking the value 1 if 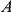 equals 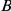 and 0 otherwise. This calculation is based on transmitted haplotypes only, and is evaluated across all haplotype explanations 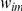. For the pseudo-control, the haplotype weight is derived similarly, based on non-transmitted haplotypes:
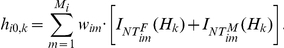
At this stage, the row vector 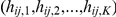 can serve as the individual's haplotype coding if all 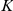 haplotypes are included for analysis.

For the example in the previous section, the haplotype coding for the pseudo-control is 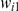 for (12), 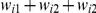 for (22), and zero for the remaining haplotypes. While for the affected child, there is no uncertainty in phase and thus the coding is 2 ( = 1+1) for haplotype (11) and zero for the rest. Again, it can be seen that 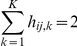, as in the case when phase is known.

### Haplotype Coding with Ancestry Uncertainty – Dimension Reduction

In the likelihood function under the conditional logistic regression model in equation (1), the design matrix 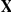 containing haplotype likelihoods 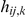 can be sparse due to the large number 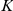 of haplotypes available, and some 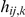 may be extremely small or zero. Instead of trimming those rare haplotypes, we adopt an evolutionary-guided procedure to merge “young” haplotypes with their “ancestors”. This clustering concept has been considered for case-control studies [10], for TDT-type tests [15], [16], and for likelihood ratio tests conducted in family studies [11]. Because rare haplotypes have a lower expected age, common haplotypes are therefore considered more ancient, and ancestral haplotypes will be defined as core haplotypes.

Suppose the number of core haplotypes 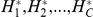 is 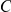, and the 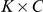 matrix 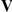 with entries 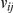 representing the probability that haplotype 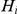 is clustered to the core 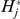. For instance, the (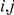)-th entry is 1 if the original haplotype 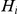 is clustered to the core haplotype 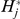, and zero otherwise. If 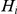 is grouped to 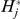 with probability 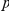, then 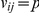. Note that every row in 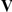 sums to 1, i.e. 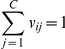. Then, the original design matrix 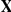 of haplotype likelihoods 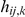 can be represented as 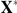 (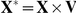) with 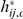 denoting the corresponding entries. This new matrix is now equipped with the uncertainty in haplotype phase, in haplotype transmission, and in ancestry clustering, and it can be shown with simple algebra that 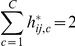.We will use this uncertainty-coding matrix in conditional logistic regression analysis later.

Following the formulation, the model becomes
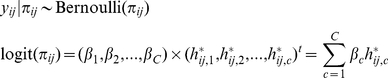
where the likelihood for the 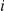-th family can be written as
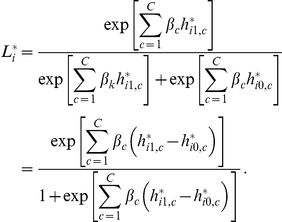
The prior distribution for the 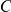-dimensional random vector 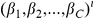 is a multivariate normal distribution with the mean vector 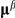 and variance covariance matrix 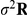,
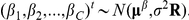
Note that the covariance matrix can be non-diagonal to account for the fact that summation of 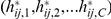 is constrained. Each component in the 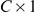 vector 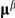 (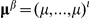) is the logit transform of prevalence of the disease under investigation. For 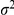, a hyper-prior inverse gamma distribution (*IG*) is assumed and 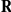 is the identity matrix if the 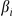
's are independent. The statistical inference will be made based on posterior samples generated from Markov chain Monte Carlo (MCMC) methods via the package BRugs in R.

### Computational Notes

The whole procedure discussed above involves (1) estimation of the haplotype frequency, (2) development of the clustering matrix 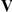, (3) evaluation of the likelihoods for haplotype explanation 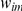, (4) construction of the matrix 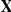, (5) computation of the final uncertainty-coding matrix 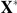, and (6) computation of the posterior sample for statistical inference. Steps (1) and (3) can be conducted in FAMHAP [17], [18], steps (2), (4) and (5) are carried out with R codes, and the final step (6) can be performed in BRugs. To complete these steps, we integrate BRugs and FAMHAP, along with our codes written in R. The whole package (called BRUCM for Bayesian Regression with Uncertainty-Coding Matrix) has been tested in the R environment and is freely available at the webpage http://homepage.ntu.edu.tw/~ckhsiao/download(en).html. In the Bayesian model specification, the prior distribution can be either user-defined or selected from the reference priors provided in the code.

## Results

### Sampling Scheme and Computation for Simulations

Simulation studies were conducted to evaluate the performance of the proposed approach and to compare it with FBAT, a procedure commonly applied in family association studies. We selected from the HapMap homepage (http://www.hapmap.org) a haplotype region containing 8 SNPs (rs2301756, rs12423190, rs11066322, rs7975439, rs7313360, rs7958372, rs3741983, and rs7953150) on 12q24 linked to metabolic syndrome. The frequencies of each SNP and phased haplotype are listed in Table 1. Note that the haplotype 11111211 with frequency 0.10 was taken as the risk haplotype. Family data were generated based on different modes of inheritance (additive, dominant, or recessive), relative risk (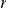 = 1.2, 1.5, or 2.0), and prevalence (0.01). The haplotypes of the affected child were first generated, then the two other haplotypes were generated to set up the parents' four haplotypes. Based on these, we could construct the haplotypes of other siblings. Each family had at least one affected child. The number of families was fixed at 200, where the number of family members in each family was 3 plus a Poisson distribution with mean at 2. Therefore, each family was guaranteed to have at least three members. About 81% of the 200 families, the number of family members was greater than 3. In total, there were nine simulation settings, and under each setting the number of replications was 1000.

**Table 1:** Frequencies (in percentages) of the simulated haplotypes and the distribution of SNPs.

	SNP composition of the haplotype								
Haplotype	S1	S2	S3	S4	S5	S6	S7	S8	Freq.(%)
11111111	1	1	1	1	1	1	1	1	49.44
12111111	1	2	1	1	1	1	1	1	27.78
11111211	1	1	1	1	1	2	1	1	10.00
21212121	2	1	2	1	2	1	2	1	7.22
21212122	2	1	2	1	2	1	2	2	3.89
12111211	1	2	1	1	1	2	1	1	1.11
11222122	1	1	2	2	2	1	2	2	0.56
MAF^a^ (%)	11.11	28.89	11.67	0.56	11.67	11.11	11.67	4.45	

a MAF for minor allele frequency.

In each replication, family genotypes were first constructed based on simulated haplotypes, then the frequencies of haplotypes were estimated and the clustering step was conducted. Following Shannon's information criterion, the original seven haplotypes were clustered to five core haplotypes. Four of the five cores were recovered in every replication, while one was recovered in 92% of the simulations. In less than 7% of all replications, this procedure identified more than seven haplotypes from the genotype data. Those were, however, rare haplotypes and did not affect the set of core haplotypes. Next, the uncertainty-coding matrix 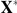 was derived based on both the clustering matrix 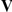 and the original design matrix 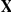. Finally, the BRugs package was called in R to generate posterior samples for Bayesian inference under the same model specified in previous sections with 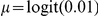 and 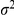 from 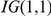. For each parameter, we disregarded the initial 5,000 iterations for burn-in, and we collected every tenth value in the following 10,000 runs to reduce the correlation between samples in each of three chains. This led to 3,000 posterior samples.

### Performance Evaluation

**Figure 1:**
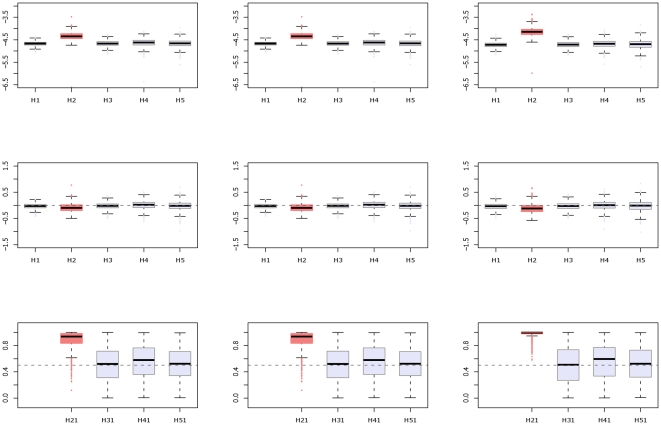
Boxplots of haplotype effects under additive models. Boxplots of 1000 replications for additive model under 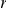 = 1.2 (1st column), 1.5 (2nd column) and 2.0 (3rd column). The first row contains posterior mean effects of 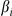, the second is for its bias, and the last is for the posterior probability of susceptibility 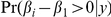. Red plots correspond to the risk haplotypes.

To evaluate the performance of this procedure, we examined the posterior mean effect 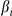, the risk relative to the most common haplotype 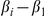, and the posterior probability of susceptibility 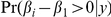. Figure 1 displays the boxplots of 1000 replications for the additive model under 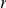 = 1.2, 1.5 and 2.0. The first row shows that the haplotype 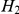 is predominantly identified as the higher risk haplotype. The second row shows the bias of the estimated effects, and the bottom row shows that the posterior probability of susceptibility can be as high as 0.71 for 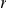 = 1.2, and 0.96 for 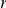 = 2.0. Plots for other modes of inheritance are provided in Figures S1 and S2.

**Figure 2:**
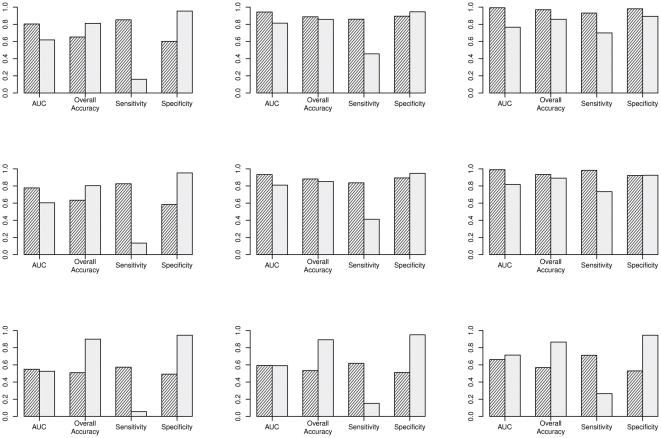
Performance evaluation under different genetic models and relative risk ratios. The performance evaluation based on AUC, overall accuracy, sensitivity, and specificity. The three columns are results under 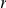 = 1.2, 1.5, and 2.0, respectively. The three rows are simulations from additive (top), dominance (middle), and recessive models (bottom), respectively. The shaded bars in the left are under the hierarchical model with independent priors on regression coefficients, and the right bars contain results from FBAT.

As a comparison with FBAT, we calculated sensitivity, specificity, overall accuracy, and area under the ROC curve (AUC) for each simulation setting with the Bayesian procedure and FBAT, respectively. In each replication, the haplotype was identified as a risk factor if its posterior probability of positive relative risk 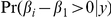 was greater than 50%. In addition, the sensitivity and specificity for determination of risk and non-risk haplotypes were computed. The overall accuracy was calculated as the percentage of correct classification of the haplotypes as risk or non-risk, while the AUC was derived by varying the threshold value T in the posterior probability 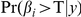. Figure 2 shows the sensitivity, specificity and the corresponding overall accuracy on the ROC curve under the Bayesian model, along with the significance tests from FBAT. FBAT tended to have high specificity, leading to high overall accuracy. However, when looking at the AUC and sensitivity, Bayesian analysis provided better and more stable results, except under the recessive model where all procedures failed to perform satisfactorily. Detailed results and numbers are listed in Table 2.

**Table 2:** Performance comparison between BRUCM and FBAT.

	BRUCM			FBAT		
	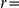 1.2	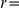 1.5	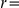 2.0	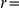 1.2	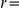 1.5	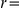 2.0
AUC						
Additive	0.804	0.943	0.992	0.618	0.813	0.766
Dominant	0.778	0.933	0.990	0.604	0.810	0.818
Recessive	0.549	0.593	0.662	0.525	0.591	0.713
Overall Accuracy						
Additive	0.652	0.887	0.970	0.811	0.857	0.858
Dominant	0.635	0.881	0.934	0.804	0.851	0.891
Recessive	0.510	0.534	0.568	0.900	0.892	0.866
Sensitivity						
Additive	0.852	0.859	0.931	0.160	0.455	0.700
Dominant	0.826	0.837	0.982	0.135	0.412	0.734
Recessive	0.573	0.618	0.712	0.055	0.152	0.266
Specificity						
Additive	0.601	0.895	0.980	0.954	0.945	0.893
Dominant	0.585	0.894	0.922	0.952	0.947	0.925
Recessive	0.492	0.511	0.529	0.945	0.950	0.946

Performance comparison between Bayesian regression with uncertainty-coding matrix (BRUCM) and FBAT under independent prior distributions on 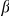
's, genotype relative risks 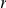, and modes of inheritance.

### Application: Taiwan Schizophrenia Linkage Study

**Figure 3:**
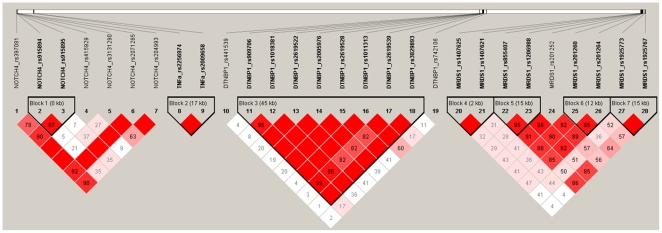
LD information for the schizophrenia study. LD blocks of the 28 SNPs on chromosome 6p for the schizophrenia multiplex family study. The genotype data from the largest block (3rd block) were selected for analysis.

Schizophrenia is a disabling mental disorder with a lifetime risk of 0.72% worldwide [19], and many studies have identified the association between schizophrenia and genetic/environmental factors [20], [21]. Two studies, the Taiwan Schizophrenia Linkage Study [22] and the Multidimensional Psychopathological Study on Schizophrenia [23], have collected multiplex family data for analysis. The first study recruited schizophrenic patients and their first-degree relatives, whereas the second study recruited sib-pairs who were both affected and their first-degree relatives [22]–[24]. This data set contains the genotyping information on chromosome 6p of 1016 individuals from 218 multiplex families. Among them, ninety-three families had two offspring, 108 families had three, and 17 families had four or five offspring. Twenty-eight SNPs were genotyped, which cover 4 genes: *MRDS1*, *DTNBP1*, *TNFα*, and *NOTCH4*. After performing haplotype block construction with linkage disequilibrium (LD), the largest block, the third one, was selected for analysis (Figure 3). This block belongs to *DTNBP1* gene, and contains, in order, the 8 SNPs rs909706 (P1583), rs1018381 (P1578), rs2619522 (P1763), rs2005976 (P1757), rs2619528 (P1765), rs1011313 (P1325), rs2619539 (P1655), and rs3829893 with corresponding common/minor alleles T/C, C/T, A/C, G/A, G/A, C/T, C/G, and G/A. There were 12 haplotypes in total, 8 of which were rare with frequency less than 5% (Table S1). The number of resulting core haplotypes was 5 based on Shannon's criterion (see cladogram in Figure S3), and the corresponding revised frequencies are listed in Table S1, along with the original haplotype composition and estimated frequencies derived by FAMHAP. The summation of frequencies of these 5 core haplotypes is 98.95%. Next, the matrices 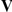 and 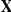 were constructed to form the design matrix 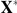 for further Bayesian analysis.

The complete model specification is
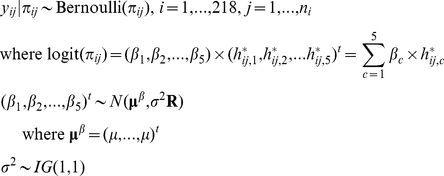
Note that each component 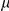 in the 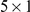 mean vector 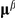 was fixed at logit(0.72%) and *IG* stands for the inverse Gamma distribution. The MCMC computational method in BRugs was applied, and the trace plot was inspected. The sampler mixed well and the resulting Gelman-Rubin convergence diagnosis measure was 1. The initial 30000 iterations were burn-in and every 60th value was kept as a sample. A total of 1500 samples were used for posterior analysis and the effective sample size for key parameters ranged from 982 to 1500.

**Figure 4:**
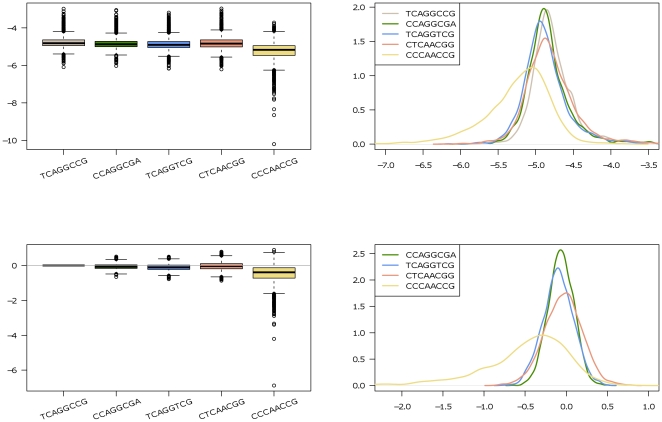
Boxplots and posterior density plots for the schizophrenia study. Boxplots and density plots of the posterior distributions of 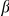
's (top two plots) and 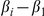 (bottom two plots) for schizophrenia study.

Figure 4 shows the boxplots and posterior density plots of the haplotype-specific effects 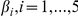, and the relative effects 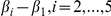, respectively. Note that, except for the fifth haplotype, the other four (TCAGGCCG, CCAGGCGA, TCAGGTCG, and CTCAACGG) seem to share similar risk. The density corresponding to the fifth haplotype locates the farthest left in Figure 4 (in the upper right panel), indicating a comparatively high protective effect with a posterior probability of only 0.15 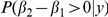 (Table 3). This implies a smaller relative risk associated with this haplotype, as compared with that of the other four, which all show similar values close to 0.5. In FBAT, however, rare haplotypes, i.e. those present in less than 10% of all families included, cannot be tested and thus no conclusion can be made about the marginal or relative risks of the last haplotype (last column in Table 3).

**Table 3:** Summary statistics for the schizophrenia study.

Core haplotype		Posterior		FBAT	
No.	Configuration	Mean(sd)	Postr. RR	Score	P-value
1	TCAGGCCG	−4.77(0.31)	-	−2.08	0.80
2	CCAGGCGA	−4.83(0.31)	0.34	−2.12	0.78
3	TCAGGTCG	−4.87(0.31)	0.30	5.39	0.39
4	CTCAACGG	−4.81(0.34)	0.44	1.81	0.69
5	CCCAACCG	−5.25(0.47)	0.15	-	-

Posterior means (Mean) and standard deviations (sd) are for the core haplotype effects, while posterior probability 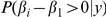 is relative to the most common haplotype 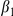 (under Postr. RR). The last two columns contain results (Score and P-value) from FBAT.

## Discussion

In family studies with collected genotype data, the inference of haplotype risk requires the determination of haplotype phase and corresponding transmission and non-transmission status. This task becomes even more complicated when the number of haplotypes is large and when some of them are of small frequencies. In this paper, we first constructed clusters of haplotypes based on their evolutionary relationship to reduce dimension of parameters, and then combined this cluster structure with the haplotype phase and transmission uncertainty to derive an uncertainty-coding matrix. This matrix was next used in a Bayesian conditional logistic regression model to examine the existence of haplotype risk. This proposed approach not only provides a probabilistic risk evaluation for haplotypes under study, it also integrates into the analysis the variability from various sources and reduces successfully the number of haplotypes involved in the genomic region.

The proposed approach has several strengths. First, this clustering design is good for the case where several evolutionary-related variants contribute similarly to the disease association. For instance, when one core haplotype is estimated with a high posterior probability of risk, it may imply that the rare haplotypes being clustered with it share similar and possibly minor risk as well. In other words, this “core cluster” may represent a homogeneous group worthy of further investigation in association studies. The proposed methodology may be applied under the assumption of common disease rare variants (CDRV), especially when these rare variants are related in the evolutionary sense. That is, the core set of such clustered haplotypes may explain better the association between disease and markers. It should be kept in mind, however, that this current approach cannot identify the risk of each rare haplotype in the same group, unless more subjects with such haplotypes can be collected.

A second strength is that such a regression model can be easily extended to include other clinical information or environmental covariates for examination of genetic and environmental interaction. Taking the schizophrenia study for example, other research has reported the importance of negative symptoms [25]. The inclusion of scores from questionnaires about negative symptoms or other clinical features of schizophrenia may clarify the role *DTNBP1* plays in brain function in schizophrenic patients. A third strength is the ability to incorporate haplotypes from other genomic regions so that the joint effect and interactions of haplotypes locating in different genes can be assessed simultaneously. Suppose 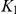 and 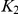 are numbers of haplotypes in two different regions, then the number of parameters can be reduced from 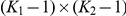 to 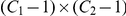 for the evaluation of joint effects, where 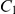 and 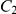 are numbers of corresponding core haplotypes in each region, respectively.

The debate of association between *DTNBP1* and schizophrenia has not been settled and as of yet no global significance has been identified [26], [27]. Although the last haplotype (CCCAACCG) shows effects different from the remaining core haplotypes, their effect sizes are all too similar to reach a definitive conclusion. In addition to the possible explanations listed in previous studies, here we suggest focusing on the fourth and fifth haplotypes, because their descendant haplotypes overlap. Our current approach assumes all haplotypes in the same core set contribute equally to the disease association. This assumption, however, may fail in the case where disease susceptibility exhibits etiological heterogeneity. In other words, the original haplotype construction based on “haplotype blocks” may need further examination. This methodological issue and development will be incorporated in future studies.

The schizophrenia multiplex family study originally considered 12 haplotypes, which were then clustered into 5 core haplotypes. This reduction (from 12 to 5) may not be impressive in terms of number of parameters and computational burden. Therefore we have included another study about Crohn's disease in Supporting Information Text S1 where 27 haplotypes are clustered to 6 core haplotypes. The reduction in this case is much more substantial, and our proposed methodology also offers an evolutionary interpretation and provides a solution to collinearity. Without this reduction, the large number of parameters could lead to failure of convergence in estimation procedures in regression models.

One issue with regard to the Bayesian approach concerns the choice of prior distributions. Analysis of the sensitivity of the posterior inference to the prior specification can help evaluate the influence of this choice. We have considered both independent and correlated priors, and both conjugate beta and non-informative truncated normal distributions in the analysis. Their AUC, overall accuracy, sensitivity, and specificity are similar and the general conclusions do not differ (data not shown). These findings indicate that the posterior inference is not sensitive to the prior considered. Special care needs to be taken, however, in the choice of prior mean for the regression coefficient 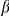 for the haplotypes. The mean should reflect properly current knowledge of the disease and we recommend using the logit transform of disease prevalence for the prior mean 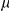 to expedite convergence in computations. The proposed approach may look complicated at first. Fortunately, several steps can be done with help from currently available algorithms. In addition to the code we have developed, our proposal integrates the clustering algorithms in Tzeng [10] and Lee et al. [11], the likelihoods of haplotype configurations from FAMHAP [17], [18], and Bayesian analysis with the BRugs function in R. The proposed procedure, as well as the computation of the uncertainty-coding matrix, has been implemented, and the codes are freely available for download.

Alternatively, after the uncertainty-coding matrix is constructed, one may pursue non-Bayesian analysis, such as LASSO and ridge regularized regression to handle the collinearity problem in the design matrix 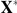 [28]. Such regularized regression models impose a penalty 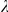 on regression coefficients 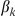 (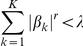, where 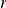 = 1 or 2) and obtain biased estimates with reduced variance. This regularized technique has been applied to high-throughput microarray data for quantitative disease phenotypes, and the inclusion of the uncertainty-coding matrix should not give rise to any further difficulty. When binary disease status is of interest, however, extra care needs to be taken and this warrants further study.

### Web Resources

The URL for the program (called BRUCM) written in R is http://homepage.ntu.edu.tw/~ckhsiao/download(en).html

## Supporting Information

Figure S1
**Boxplots of haplotype effects under dominance models.** Boxplots of 1000 replications for dominance model under 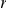 = 1.2 (first column), 1.5 (second column) and 2.0 (third column). The first row contains posterior mean effects of 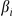, the second row is for its bias, and the last row is for the posterior probability of susceptibility 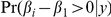. Red plots correspond to the risk haplotypes.(TIF)

Figure S2
**Boxplots of haplotype effects under recessive models.** Boxplots of 1000 replications for recessive model under 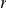 = 1.2 (first column), 1.5 (second column) and 2.0 (third column). The first row contains posterior mean effects of 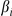, the second row is for its bias, and the last row is for the posterior probability of susceptibility 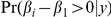. Red plots correspond to the risk haplotypes.(TIF)

Figure S3 **The cladogram of 12 haplotypes in the third block for the schizophrenia study.**
(TIF)

Table S1 **Summary statistics for the schizophrenia study.** Frequencies are for the original haplotypes (Before) and haplotypes after grouping (After).
(DOC)

Text S1 **Analysis of Crohn's Disease data based on 6 core haplotypes.**
(PDF)

## References

[1] Schaid D, Rowland C, Tines D, Jacobson R, Poland G (2002). Score tests for association between traits and haplotypes when linkage phase is ambiguous.. American Journal of Human Genetics.

[2] Zaykin D, Westfall P, Young S, Karnoub M, Wagner M (2002). Testing association of statistically inferred haplotypes with discrete and continuous traits in samples of unrelated individuals.. Human Heredity.

[3] Mensah FK, Gilthorpe MS, Davies CF, Keen LJ, Adamson PJ (2007). Haplotype uncertainty in association studies.. Genetic Epidemiology.

[4] Horvath S, Xu X, Lake SL, Silverman EK, Weiss ST (2004). Family-based tests for associating haplotypes with general phenotype data: Application to asthma genetics.. Genetic Epidemiology.

[5] Purcell S, Daly MJ, Sham PC (2007). WHAP: haplotype-based association analysis.. Bioinformatics.

[6] Sham P, Rijsdijk F, Knight J, Makoff A, North B (2004). Haplotype association analysis of discrete and continuous traits using mixture of regression models.. Behavior Genetics.

[7] Morris AP (2005). Direct analysis of unphased SNP genotype data in population-based association studies via Bayesian partition modelling of haplotypes.. Genetic Epidemiology.

[8] Cordell HJ (2006). Estimation and testing of genotype and haplotype effects in case-control studies: comparison of weighted regression and multiple imputation procedures.. Genetic Epidemiology.

[9] Igo RP, Li J, Goddard KAB (2009). Association mapping by generalized linear regression with density-based haplotype clustering.. Genetic Epidemiology.

[10] Tzeng JY (2005). Evolutionary-based grouping of haplotypes in association analysis.. Genetic Epidemiology.

[11] Lee MH, Tzeng JY, Huang SY, Hsiao CK (2011). Combining an Evolution-guided Clustering Algorithm and Haplotype-based LRT in Family Association Studies.. BMC Genetics.

[12] Lin DY (2006). Likelihood-based inference on haplotype effects in genetic association studies.. Journal of the American Statistical Association.

[13] Soofi ES (1990). Effects of collinearity on information about regression coefficients.. Journal of Econometrics.

[14] Krishna A, Bondell HD, Ghosh SK (2009). Bayesian variable selection using an adaptive powered correlation prior.. Journal of Statistical Planning and Inference.

[15] Seltman H, Roeder K, Devlin B (2001). Transmission/disequilibrium test meets measured haplotype analysis: family-based association analysis guided by evolution of haplotypes.. American Journal of Human Genetics.

[16] Seltman H, Roeder K, Devlin B (2003). Evolutionary-based association analysis using haplotype data.. Genetic Epidemiology.

[17] Becker T, Knapp M (2004). Maximum-likelihood estimation of haplotype frequencies in nuclear families.. Genetic Epidemiology.

[18] Herold C, Becker T (2009). Genetic association analysis with FAMHAP: a major program update.. Bioinformatics.

[19] Saha S, Chant D, Welham J, McGrath J (2005). A systematic review of the prevalence of schizophrenia.. PLoS Medicine.

[20] Tsuang M (2000). Schizophrenia: genes and environment.. Biological Psychiatry.

[21] Walker E, Kestler L, Bollini A, Hochman KM (2004). Schizophrenia: etiology and course.. Annual Review of Psychology.

[22] Hwu HG, Faraone SV, Liu CM, Chen WJ, Liu SK (2005). Taiwan schizophrenia linkage study: The field study.. American Journal of Medical Genetics Part B: Neuropsychiatric Genetics.

[23] Tsuang HC, Lin SH, Liu SK, Hsieh MH, Hwang TJ (2006). More severe sustained attention deficits in nonpsychotic siblings of multiplex schizophrenia families than in those of simplex ones.. Schizophrenia Research.

[24] Lin SH, Liu CM, Liu YL, Fann CSJ, Hsiao PC (2009). Clustering by neurocognition for fine mapping of the schizophrenia susceptibility loci on chromosome 6p.. Genes, Brain and Behavior.

[25] Ross CA, Margolis RL, Reading SAJ, Pletnikov M, Coyle JT (2006). Neurobiology of schizophrenia.. Neuron.

[26] Holliday EG, Handoko HY, James MR, McGrath JJ, Nertney DA (2006). Association study of the dystrobrevin-binding gene with schizophrenia in Australian and Indian samples.. Twin Research and Human Genetics.

[27] Liu CM, Liu YL, Fann CSJ, Yang WC, Wu JY (2007). No association evidence between schizophrenia and dystrobrevin-binding protein 1 (DTNBP1) in Taiwanese families.. Schizophrenia Research.

[28] Guo W, Lin S (2009). Generalized linear modeling with regularization for detecting common disease rare haplotype association.. Genetic Epidemiology.

