# Supplementary figures and images for: Using an Uncertainty-Coding Matrix in Bayesian Regression Models for Haplotype-Specific Risk Detection in Family Association Studies
Source: PLoS One. 2011 Jul 15;6(7):e21890. doi: 10.1371/journal.pone.0021890 (PMC3137600; doi:10.1371/journal.pone.0021890)

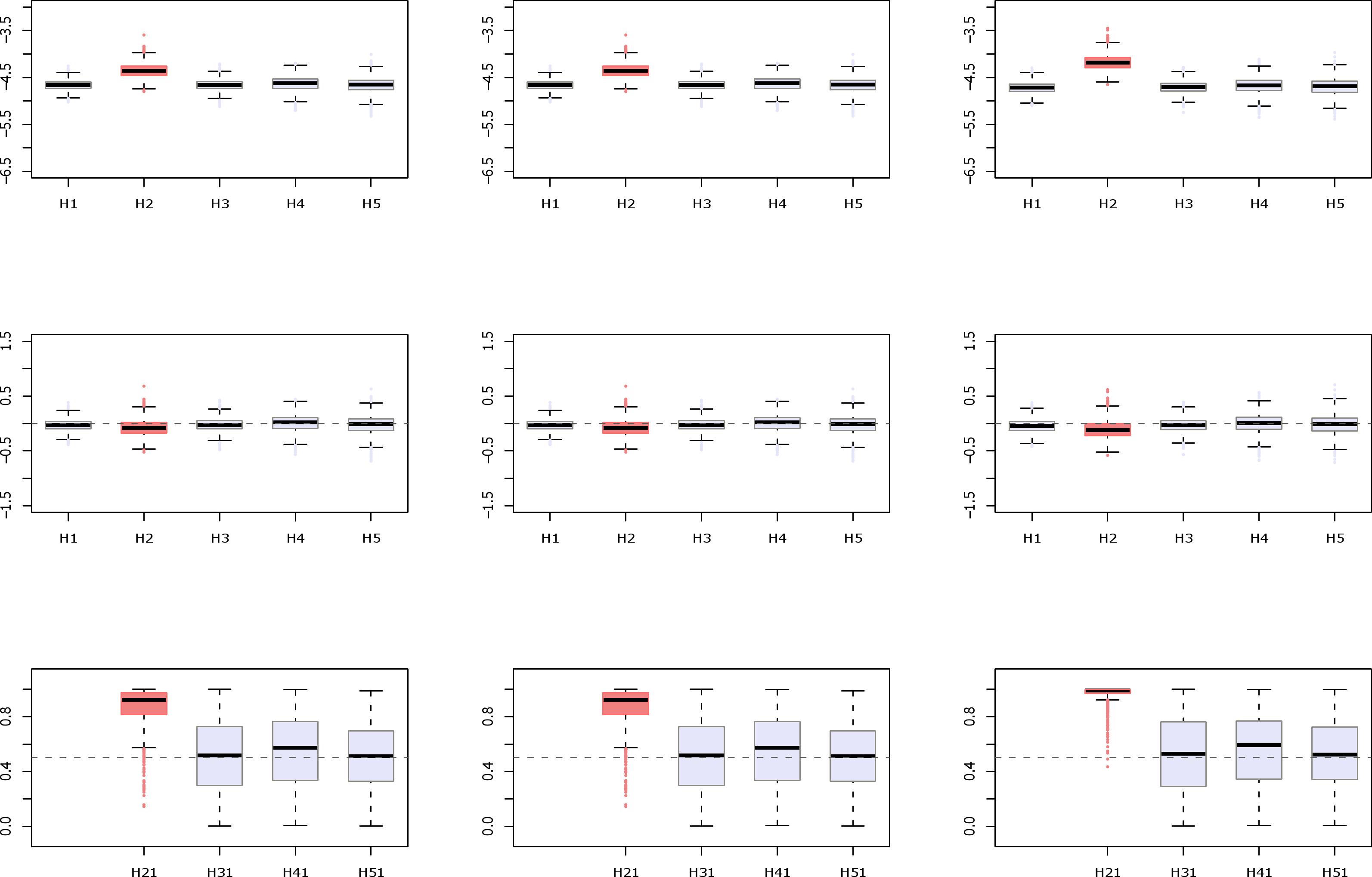

Supplement: Figure S1 — Boxplots of haplotype effects under dominance models. Boxplots of 1000 replications for dominance model under = 1.2 (first column), 1.5 (second column) and 2.0 (third column). The first row contains posterior mean effects of , the second row is for its bias, and the last row is for the posterior probability of susceptibility . Red plots correspond to the risk haplotypes. (TIF) [file pone.0021890.s001.tif]

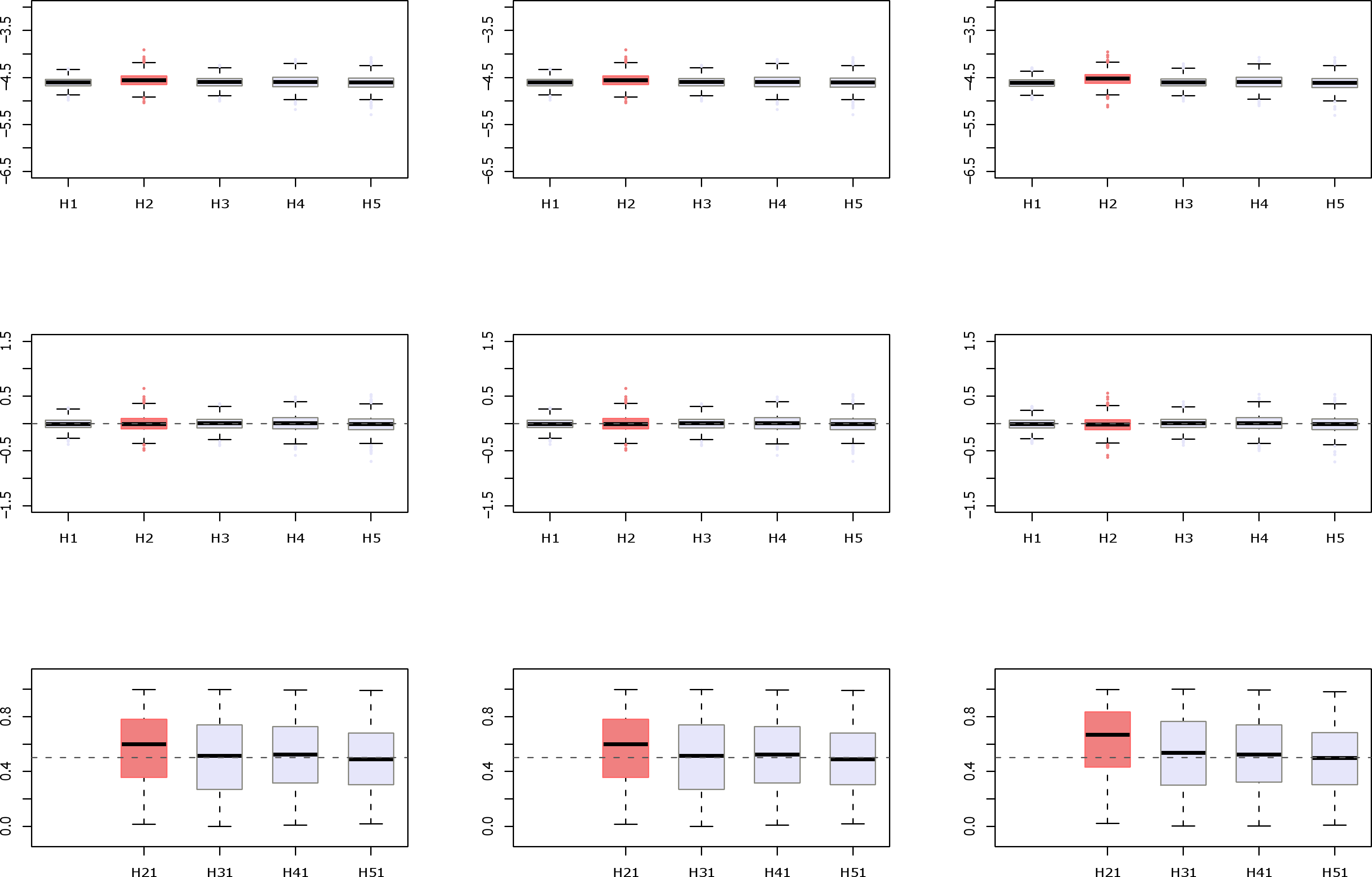

Supplement: Figure S2 — Boxplots of haplotype effects under recessive models. Boxplots of 1000 replications for recessive model under = 1.2 (first column), 1.5 (second column) and 2.0 (third column). The first row contains posterior mean effects of , the second row is for its bias, and the last row is for the posterior probability of susceptibility . Red plots correspond to the risk haplotypes. (TIF) [file pone.0021890.s002.tif]

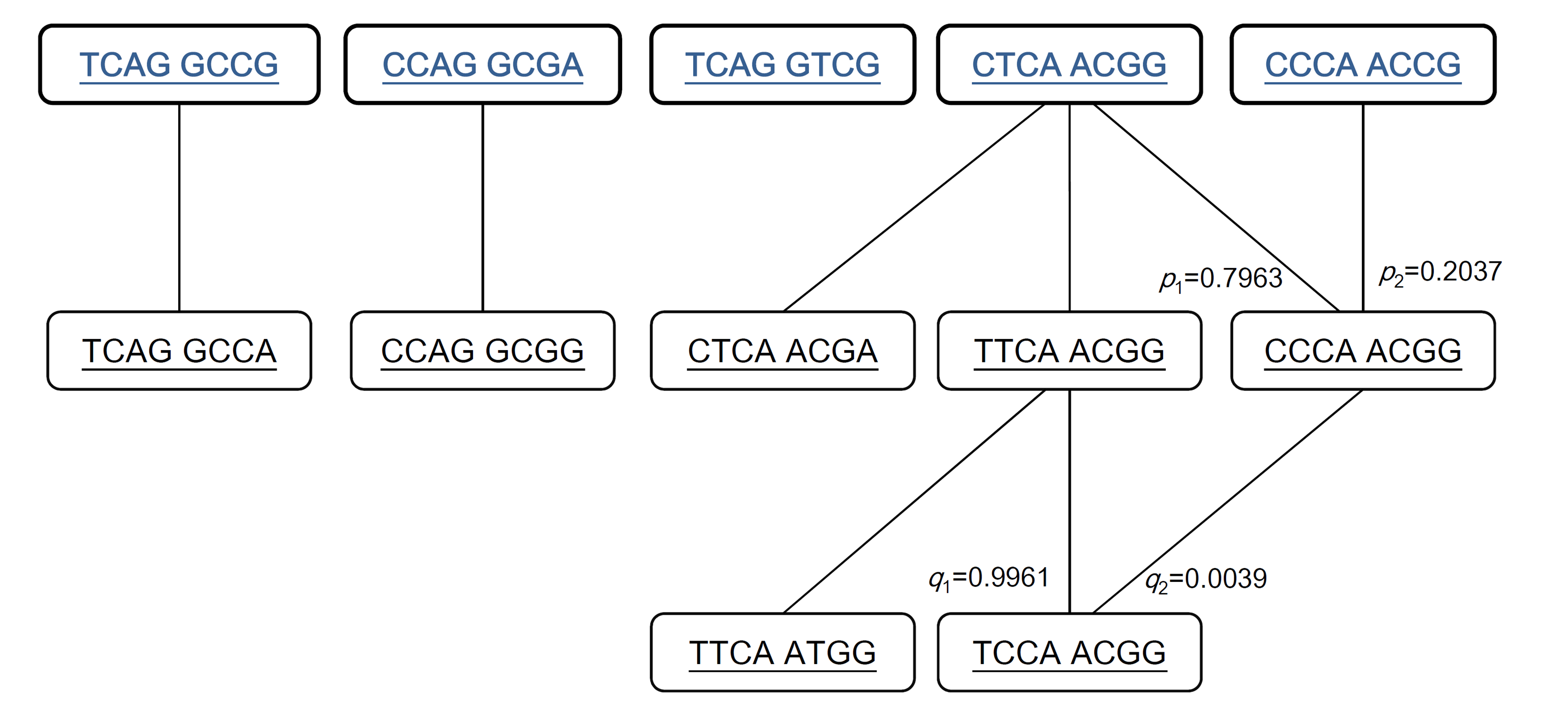

Supplement: Figure S3 — The cladogram of 12 haplotypes in the third block for the schizophrenia study. (TIF) [file pone.0021890.s003.tif]
