# Supplementary material for: Using an Uncertainty-Coding Matrix in Bayesian Regression Models for Haplotype-Specific Risk Detection in Family Association Studies
Source: PLoS One. 2011 Jul 15;6(7):e21890. doi: 10.1371/journal.pone.0021890 (PMC3137600; doi:10.1371/journal.pone.0021890)
Supplement: Table S1 — Summary statistics for the schizophrenia study. Frequencies are for the original haplotypes (Before) and haplotypes after grouping (After). (DOC) [file pone.0021890.s004.doc]

**Table S1. Summary statistics for the schizophrenia study. Frequencies are for the original (Before) haplotypes and haplotypes after grouping for the schizophrenia study.**

| **Haplotype** | |  | **Frequency (%)** | |
| --- | --- | --- | --- | --- |
| **No.** | **Form** |  | **Before** | **After** |
| **1** | TCAGGCCG |  | 40.026 | 40.288 |
| **2** | CCAGGCGA |  | 30.729 | 30.73 |
| **3** | TCAGGTCG |  | 19.199 | 19.199 |
| **4** | CTCAACGG |  | 7.165 | 7.953 |
| **5** | CCCAACCG |  | 1.833 | 1.833 |
| **6** | TTCAACGG |  | 0.262 | **－** |
| **7** | TCAGGCCA |  | 0.262 | **－** |
| **8** | CTCAACGA |  | 0.262 | **－** |
| **9** | TTCAATGG |  | 0.262 | **－** |
| **10** | CCAGGCGG |  | 0.001 | **－** |
| **11** | CCCAACGG |  | 0.001 | **－** |
| **12** | TCCAACGG |  | 0.001 | **－** |
