# Supplementary material for: Using an Uncertainty-Coding Matrix in Bayesian Regression Models for Haplotype-Specific Risk Detection in Family Association Studies
Source: PLoS One. 2011 Jul 15;6(7):e21890. doi: 10.1371/journal.pone.0021890 (PMC3137600; doi:10.1371/journal.pone.0021890)
Supplement: Text S1 — Analysis of Crohn's Disease data based on 6 core haplotypes. (PDF) [file pone.0021890.s005.pdf]

Text S1.

ANALYSIS OF CROHN'S DISEASE DATA BASED ON 6 CORE HAPLOTYPES

This family study investigated the association between 103 SNPs on chromosome 5q31 and Crohn's disease (data from http://www.broad.mit.edu/humgen/IBD5/). These SNPs clustered to 11 blocks based on levels of LD and they cover the *IBD5* gene. Previous studies identified 8 significant SNPs [1,2,3], where four of them (IGR2055a_1, IGR2060a_1, IGR2063b_1, and IGR2096a_1) locate closely in the fourth block which is composed of 11 SNPs. The allele information for the 11 SNPs is in the following table:

	rs number	SNP type
IGR2052a_1	NA[a]	C/T
IGR2055a_1	rs2248116	G/T
IGR2060a_1	rs2522057	C/G
IGR2063b_1	NA	G/C
IGR2072a_2	NA	C/T
IGR2073a_1	NA	C/A
IGR2076a_1	NA	C/T
IGR2081a_1	NA	G/A
IGR2085a_2	NA	G/A
IGR2096a_1	rs12521868	C/A
IGR2111a_3	NA	T/C

[a]: This NA indicates "rs number not assigned"

There are 27 possible haplotype compositions in this block which clustered to 6 core haplotypes based on Shannon's information criterion, as shown in the cladogram:

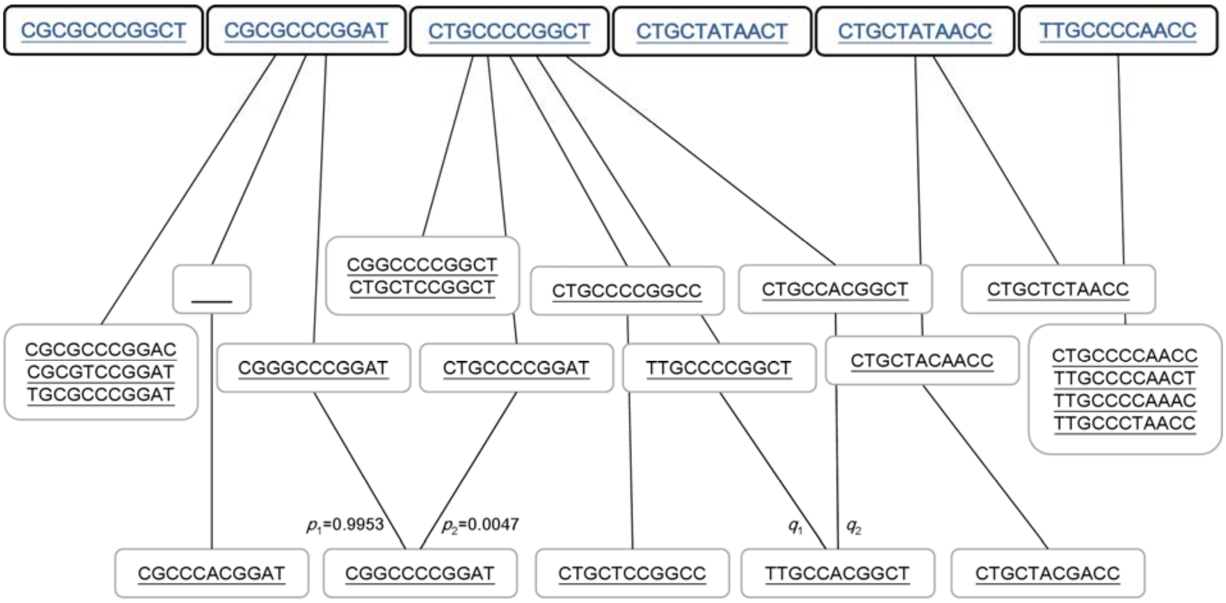

The sum of frequencies of these core haplotypes is 93.26%, and their original and revised haplotype frequencies are shown in the table.

Table: Frequencies are for the original (Before) haplotypes and haplotypes after grouping for the Crohn's disease study.

Haplotype		Frequency (%)		Haplotype		Frequency (%)	
No	Form	Before	After	No	Form	Before	After
1	CGCGCCCGGAT	33.192	35.327	15	CGCGCCCGGAC	0.394	—

2	CTGCTATAACC	23.985	24.386	16	TTGCCCCAACT	0.394	—
3	CTGCCCCGGCT	21.042	24.03	17	TTGCCCTAACC	0.394	—
4	TTGCCCCAACC	12.163	13.387	18	CTGCTCCGGCC	0.394	—
5	CTGCTATAACT	1.599	1.599	19	CTGCCACGGCT	0.228	—
6	CGCGCCCGGCT	1.275	1.275	20	CTGCTACAACC	0.207	—
7	CTGCTCCGGCT	0.899	—	21	CTGCTACGACC	0.192	—
8	CTGCCCCGGCC	0.788	—	22	TTGCCACGGCT	0.173	—
9	TGCGCCCGGAT	0.513	—	23	CGCGTCCGGAT	0.001	—
10	TTGCCCCGGCT	0.503	—	24	CGGCCCCGGCT	0.001	—
11	TTGCCCCAAAC	0.436	—	25	CTGCCCCGGAT	0.001	—
12	CGGGCCCGGAT	0.425	—	26	CTGCCCCAACC	0.001	—
13	CGGCCCCGGAT	0.405	—	27	CTGCTCTAACC	0.001	—
14	CGCCCACGGAT	0.398	—				

The results based on posterior means and the estimated risk probabilities are listed in the following table and the figure shows that the risks of these core haplotypes form two groups. The first haplotype CGCGCCCGGAT has a higher risk than other haplotypes. In this case, we select the second most common haplotype as the reference for ease of interpretation. The prior mean μ was fixed at logit(0.18%) and the prior on σ^2 was $IG(1,1)$. Relative to the reference haplotype, the posterior probability of a relatively higher risk $P(\beta_1 - \beta_2 > 0 \mid y)$ is 1. This value implies decisive evidence for this haplotype to be of high risk. In FBAT, the first haplotype was tested

with mild significance (p-value =0.053). However, FBAT considers only non-rare haplotypes and thus only four are tested.

Table. Posterior means and standard deviations are for the haplotype effects, while posterior probability $P(\beta_i - \beta_2 > 0 \mid y)$ is relative to the second most common haplotype β_2 (under Postr. RR). The last column contains results (score test and p-values) from FBAT.

Haplotype		Posterior		FBAT	
No	Form	Mean(sd)	Postr. RR	Score	p-value
1	C**GCG**CCCGG**A**T	-5.66(0.36)	1	6.5	0.053
2	C**TGC**TATAA**C**C	-6.58(0.35)	—	-2.5	0.276
3	C**TGC**CCCGG**C**T	-6.49(0.36)	0.64	-2	0.432
4	T**TGC**CCCAA**C**C	-6.29(0.37)	0.82	-1.5	0.466
5	C**TGC**TATAA**C**T	-6.51(0.53)	0.57	—	—
6	C**GCG**CCCGG**C**T	-6.38(0.51)	0.66	—	—

Figure. Boxplots and density plots of the posterior distributions of β's (top two plots) and relative $\beta_i - \beta_2$ (bottom two plots) for Crohn's disease data.

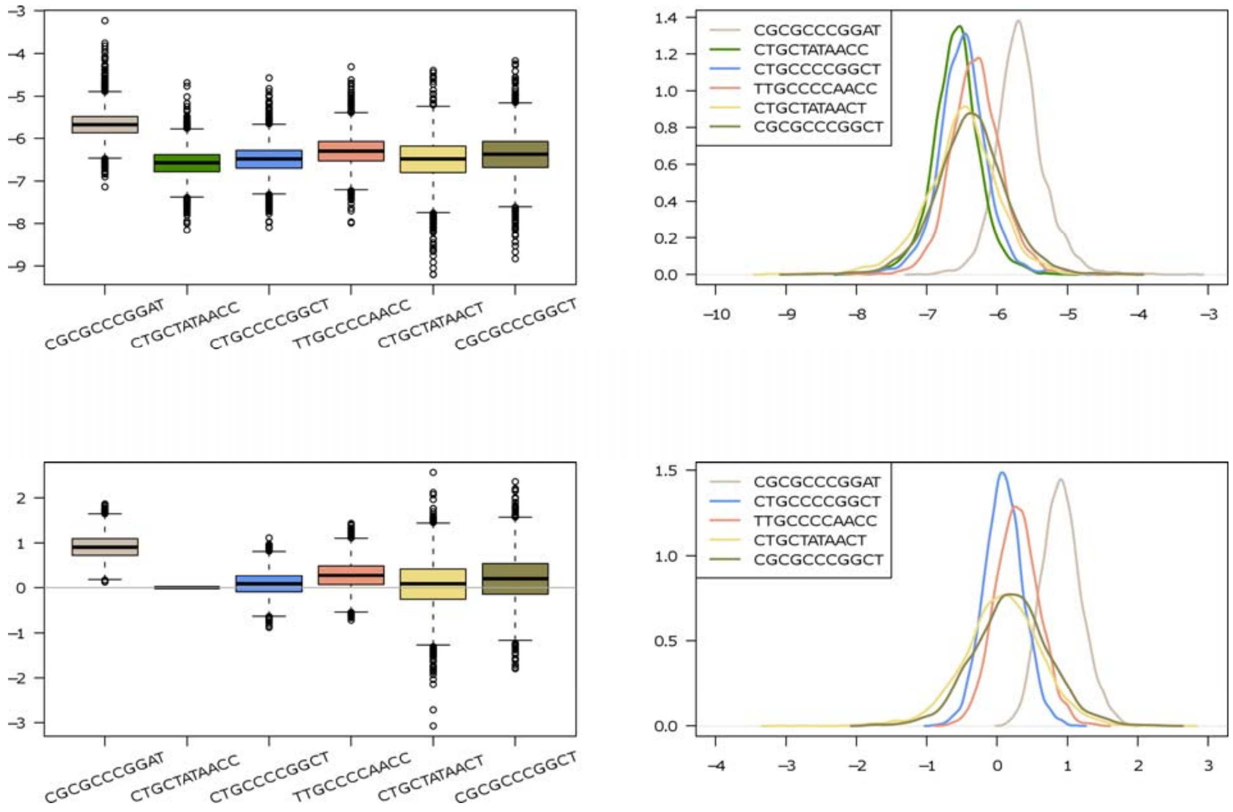

References:

1. Daly MJ, Rioux JD, Schaffner SF, Hudson TJ, Lander ES (2001) High-resolution haplotype structure in the human genome. Nat Genet 29: 229-232.

2. Rioux JD, Daly MJ, Silverberg MS, Lindblad K, Steinhart H, et al. (2001) Genetic variation in the 5q31 cytokine gene cluster confers susceptibility to Crohn disease. Nat Genet 29: 223-228.

3. Paschou P, Mahoney MW, Javed A, Kidd JR, Pakstis AJ, et al. (2007) Intra- and interpopulation genotype reconstruction from tagging SNPs. Genome Research 17: 96-107.
